# A feasibility pilot study on the use of complementary therapies delivered via mobile technologies on Icelandic surgical patients’ reports of anxiety, pain, and self-efficacy in healing

**DOI:** 10.1186/s12906-015-0613-8

**Published:** 2015-03-28

**Authors:** Margaret M Hansen

**Affiliations:** School of Nursing and Health Professions, University of San Francisco, 2130 Fulton Street, San Francisco, CA USA

**Keywords:** Complementary therapies, Relaxation techniques, Surgery, Nursing

## Abstract

**Background:**

Complementary therapies (CT), such as relaxation technique, massage, guided imagery, and accupuncture have shown to benefit patients undergoing surgery. The aim of this study was to determine the feasibility of using audio relaxation technique (ART), music intervention (MI), nature video application with music (NVAM), and nature video application without music (NVA) delivered via mobile technologies in a clinical setting. Secondary, the effects of ART, MI, NVAM and NVA on patients’ state anxiety, pain perception, and perceived self-efficacy in healing were determined.

**Methods:**

A randomized clinical trial (RCT) involving 105 same day surgery (SDS) patients, who were assigned to an ART (*n = 25*)*,* MI (*n = 25*), NVAM (*n = 15*)*,* NVA (*n = 16*)*,* or a control group (*n = 24*) were assessed for state anxiety, self-reported pain, and self-efficacy four days prior to surgery, immediately prior and following a surgical intervention, and day five post-operative.

**Results:**

ANOVA found no statistically significant differences in anxiety scores; pain, or perceived self-efficacy between the five groups. Matched pairs *t*-Test revealed all participants had an increase in anxiety from pre-op to day 10 follow-up; a significant change in pain levels from pre-op to day 10 follow-up; and all participants had a significant increase in general self-efficacy from pre-op to day 10 follow-up. Mean pain level scores from day 1 to pre-op showed a significant decrease in pain for the ART group and NVAM group. Matched pairs *t*-Test for self-efficacy scores indicated the MI group and the NVA group had significant increases in self-efficacy. A significant decrease in anxiety from pre-op to day 10 for participants reporting a prior history of anxiety and for those reporting prior history of taking anti-anxiety medications.

**Conclusions:**

Despite the non-significant findings between the five groups, at any measurement point, there were valuable trends toward significance and confirmed feasibility in a clinical setting. Among the groups there were statistically significant findings for all interventions on anxiety, pain, and self-efficacy. The feasability of the implementation of novel interventions of NVAM and NVAM adds to clinical practice and the CT literature.

**Trial registration:**

ClinicalTrials.gov Identifier: NCT02236455 (September 4, 2014)

## Background

Individuals preparing to undergo a SDS are frequently anxious and fearful of the unknown, which may have delitirous physiological effects, such as increased perceptions of acute pain and lowered self-efficacy in healing [1-4]. Irrespective of the surgical procedure, patients report fear of being unconscious, the actual surgery itself, pain associated with surgery, and the risk of dying. Moreover, pain medications or sedatives may decrease the patients’ self-efficacy in positively caring for themselves and the overall capacity to heal in a timely manner [5]. The emotional distress associated with surgery may prolong surgical procedural time and, undue technical problems may put the patient at risk for untoward risks [4]. Therefore, it is critical to create health interventions which complement traditional medicine; are easy to access and use; and, are simultaneously educational for future use.

Wellness and healing of the whole person in an integrated manner: mind, body, and soul is a focus of complementary medicine (CM). Rigorous research conducted over the past decade illustrate significant outcomes supporting the use of CT for palliative care [6], type-2 diabetes, cardiovascular disease [7], anxiety and pain reduction [1-4,8], spirituality, fatigue, and quality of life topics [9]. Music, as a nursing intervention, for postoperative pain and anxiety reduction is well documented [1-4,10,11]. Furthermore, music and sound are known for their healing powers and are considered one of the earliest forms of medicine [12]. Aluede reported music therapy in traditional African societies and elaborated on who conducted the therapy and for what type of illnesses it was used [13]. Therefore, music has a rich anthropological history in the role of healing. Relaxation technique used pre- and post-surgery has been investigated and found to be instrumental in the reduction of pain [14,15]. Novel ideas surrounding the incorporation of nature (Ecotherapy) as a health intervention for overall well-being, healing, and restoration, whilst also influencing spirituality are well documented [8,9,16,17]. Today there is more of a delineation between illness and the health/well-being paradigms. CM plays a positive role in cultivating health/well-being and shines a light on the fact health is not merely the absence of disease.

Whilst the use of mobile technologies (MT) for health promotion is becoming more prevalent and perceived to be an effective way to deliver patient care and education, there is limited concrete empirical evidence whether and how MT significantly improve patient health outcomes [18]. Furthermore, the combination of CT with MT is a new approach to health care delivery and may be a way to decrease pre- and post-operative angst and improve the overall healing process in a convenient manner.

### Conceptual framework

Based on Orem’s Self-Care [19] theory, four self-care CT delivered via MT were designed as interventions for this study knowing SDS patients’ experience state anxiety, pain, and a potential decrease in self-efficacy. Orem [19] purports individuals have an innate desire to perform self-care, maintain independence, and are a complete system functioning at a biological, social, and psychological level. The CM tenets of mind, body, and soul as being connected dovetail with Orem’s theory of self-care because both support an individual’s initiation and performance of actions in order to maintain health and well-being. Orem’s self-care theory may be used as a framework when instructing patients on how CM plays a role in regulating human psycho physiological modes of functioning [19]. Since MT have become more mainstay and are convenient to use, it was hypothesized the devices delivering the CM, such as ART, MI, NVAM, and NVA may enhance patients’ self-care capabilities if MT are afforded.

### Purpose

The aim of this pilot RCT was to identify the feasibility of using these novel mobile devices in a clinical setting and to compare the effectiveness of ART, MI, NVAM, and NVA delivered via Apple**©** iPods and iPads compared with no CT (control group) on Icelandic SDS patients’ (N = 105) day 1 baseline (four days prior to surgery), pre-, post-operative (PO), and day 10 follow-up (five days PO) for state anxiety using the State Trait Anxiety Inventory (STAI) [20]; perceived pain level using the Numeric Rating Scale (NRS) [21]; and perceived self-efficacy in healing capability measured by the General Self-Efficacy scale (GSE) [22]. Specifically, the researcher aimed to answer these questions: 1) Are there differences in SDS patients’ state anxiety, self-reported pain, and perceived self-efficacy levels in healing between the intervention and control participants pre- and PO? 2) Are there differences in state anxiety, self-reported pain levels, and perceived self-efficacy at day 1, pre-op, PO, and day 10 among the different groups? 3) Do prior self-reported characteristics of anxiety and pain have an influence on participants’ measured anxiety and pain at different times during the study? The importance of this study is to illustrate the novel use of MT to deliver CT for SDS patients as a convenient approach to assist individuals in coping with anxiety, pain, and self-efficacy.

## Methods

### Sample and setting

**Figure 1:**
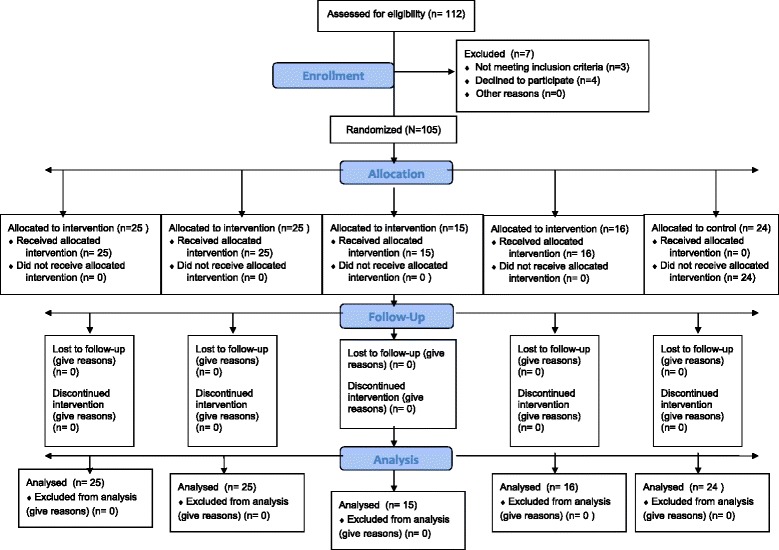
**Consort chart.**

The sample of this experimental RCT was drawn from a homogenous population of patients (N = 112) scheduled for SDS at two public hospital wards in Iceland. Of the 112 patients contacted, seven patients were excluded and 105 were randomized (see Figure 1). Two rounds of participant enrollment took place: 1) Round one consisted of 51 general surgery patients (30 females; 21 males; mean age 50.6 years (SD ±14.9) on a 21-bed SDS ward (13-D), specializing in abdominal and urinary track surgeries, from mid March to early June, 2012; 2) Round two consisted of 54 female gynecological surgical patients, mean age 42.4 years (SD ±12.5), on a 22-bed SDS ward (21-A), specializing in laparoscopy, hysteroscopy, cervical conization, and endometrial abrasion surgeries, from September to December, 2012. Inclusion criteria consisted of: age range 18-75-years; English or Icelandic speaking; cognitively alert and oriented to person, place, time, and situation; and intact visual and auditory senses. The exclusion criteria consisted of: history of substance abuse; chronic pain lasting more than six months; use of narcotic medication for more than six months; major psychiatric disorders; taking prescribed medications for thought disorders; having ophthalmology and/or auditory surgery or impairments. Five Icelandic Registered Nurses (RN), fluent in Icelandic and English, and employed on the aforementioned SDS wards, were trained to be Research Assistants (RA) by the English speaking principle investigator (PI) before the commencement of the study.

### Intervention

All participants received standard surgical care pre-, intra-, and post-operatively according to standard Icelandic clinical practice. The participants were randomized to one of five groups: audio relaxation technique (ART), music intervention (MI), nature video application with music (NVAM), and nature video application without music (NVA), or a control group. The RA telephoned all available participants and arranged to meet the intervention participants four days prior to the surgical date in order to: explain the study and answer questions; collect signed consent form and demographic data questionnaire; administer the three outcome variables as baseline; and instruct the use of the intervention which was provided via an Apple iPod **©** or iPad**©**. The mobile devices were prepared and locked up prior to the home visits. After the participant completed the baseline questionnaire the mobile device was opened and the treatment revealed. The randomization varied because there were less iPads then iPods. Upon admission to the SDS ward, the previously telephoned control group participants signed the consent form, filled out the demographic questionnaire and three outcome measurements, and had the opportunity to ask questions about the study. Each participant in the five groups had access to all pharmacological agents (e.g. Paracetamol or Ibuprofen pre-operatively; Toradol, Ketobemidone, or Morphine post-operatively), as well as non-pharmacological agents (e.g. visitors).

**Figure 2:**
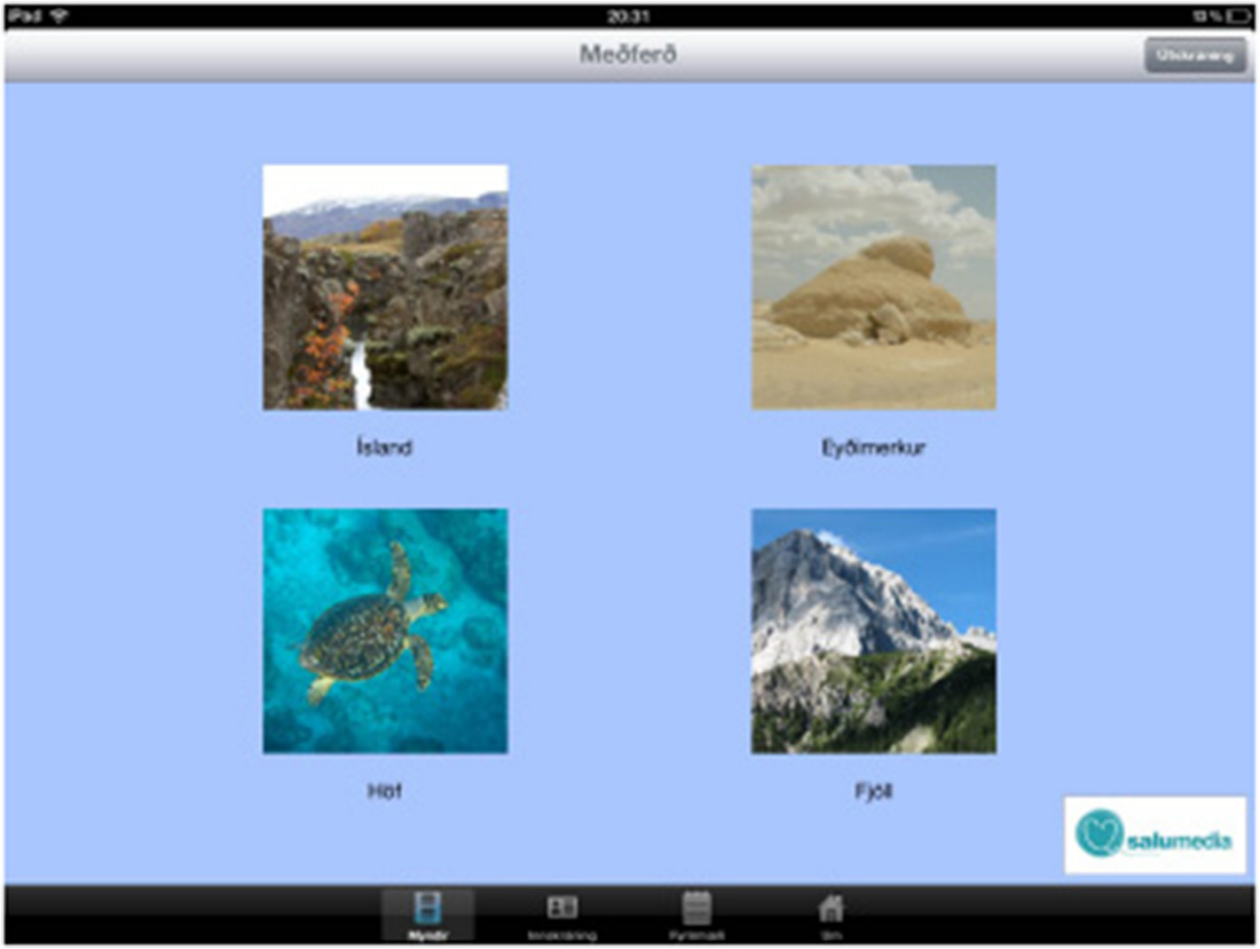
**Nature video application screen shot.** Permission to publish from Salumedia Tecnologías S.L.

The ART intervention was recorded by an Icelandic RN in Icelandic and was delivered via an iPod [23]. Permission to use the ART was obtained from the RN, before the commencement of the study. The MI was a collection of non-lyrical musical pieces, selected by the PI based upon prior studies [24] (e.g. Native American flute ensemble; Bollywood Buddha Indian music; and Shakuhachi Sakano bamboo flute music), and delivered via an iPod. Based upon Ectotherapy [17] and current literature regarding the effects of nature on the human brain [16], the PI in conjunction with multimedia designers (Salumedia.com) created nature videos that consisted of scenes of the desert, mountains, ocean, and the Icelandic wilderness with and without music (see Figure 2). Participants assigned an intervention was instructed by the RA to listen and/or view the intervention twice a day, for a minimum of 15-minutes on each occasion, each of the four days prior to the surgical date, and each of the five days following discharge from the hospital until day 10 follow-up. The participants were instructed to keep written track of the time spent on listening or viewing the intervention, however due to a lack of consistent recorded data for all participants, the time-on-intervention data were not used as a correlational variable. On the day of surgery, the participants listened/viewed the intervention pre- and post-operatively for whatever time allowed.

### Outcome variables

All outcome instruments were translated by an official translator from English to Icelandic and then checked back to English in order to assure validity of the translated forms. A demographic questionaire was developed by the PI during the design of the study and the participants’ characteristics by group are illustrated in Table 1.

**Table 1:** **Demographic characteristics by group**

*Group	**ART**	**MI**	**NVAM**	**NVA**	**Control**
Total (N = 105)	n = 25	n = 25	n = 15	n = 16	n = 24
Gender	Males = 6	Males = 4	Males = 4	Males = 2	Males = 5
	Females = 19	Female = 21	Females = 11	Females = 14	Females = 19
Age Mean (SD)	45.2 (13.4)	46.0 (15.0)	43.9 (13.5)	44.6 (16.5)	51.2 (13.7)
Past Medical History	Anxiety = 5	Anxiety = 7	Anxiety = 5	Anxiety = 4	Anxiety = 6
	Depression = 4	Depression = 5	Depression = 6	Depression = 3	Depression = 4
	Acute pain = 1	Acute pain = 2	Acute pain = 1	Acute pain = 0	Acute pain = 3
	Chronic pain = 3	Chronic pain = 4	Chronic pain = 5	Chronic pain = 3	Chronic pain = 5
Routine Medication	Anxiety = 3	Anxiety = 6	Anxiety = 3	Anxiety = 4	Anxiety = 4
	Pain = 4	Pain = 5	Pain = 4	Pain = 2	Pain = 7
Used CM	Yes = 7	Yes = 9	Yes = 3	Yes = 7	Yes = 7
Used ART	Yes = 13	Yes = 11	Yes = 4	Yes = 7	Yes = 10
Used MI	Yes = 11	Yes = 5	Yes = 2	Yes = 3	Yes = 3

*Note.* ART = audio relaxation technique; MI = music intervention; NVAM = nature video with music; NVA = nature video with music. *Both rounds combined.

Participants in the intervention groups completed a demographic survey on day 1 (four days prior to surgery) of the study, whereas control group participants completed the same demographic survey upon admission to the SDS ward on the day of surgery.

Since the aim was to assess “state anxiety,” which is considered to be a temporary fear, discomfort, or nervousness associated with a perceived situational threat (e.g. surgery), the “state” aspect of the State Trait Anxiety Inventory (STAI-Form Y) was used in this study. The “state” version (SAI) consists of 20 questions including items, such as: “I am tense; I am worried;” and “I feel calm; I feel secure.” The items are rated on a 4-point scale: “Almost never (1)” to “Almost Always (4).” Higher scores indicate higher anxiety [20]. According to the results of a prior study involving urological patients by Quek et al. [25], each of the STAI 40-items demonstrated a high degree of internal consistency with Cronbach’s alpha value = 0.38 to 0.89, whereas the Cronbach’s alpha for the same study’s total scores was 0.86. Also, the test-retest correlation coefficients for the 40-items used in the study [25] were highly significant. All participants in this study assessed pain levels using the self-report Numeric Rating Scale (NRS), which is a universal scale from 0 (no pain) - 10 (intense pain), widely used in primary care settings. Accuracy of the NRS has been reported by Krebs, Carey, and Weinberger [26]. Participants’ general self-efficacy in overall healing capability was assessed in this study by using the General Self-Efficacy (GSE) instrument consisting of 10 questions with a four-item response format (1 = Not at all true; 2 = Hardly true; 3 = Moderately true; 4 = Exactly true). Schwarzer and Jerusalem [22] report the GSE Cronbach’s alphas, derived from samples spanning 23 nations, ranged from .76 to .90, with an overarching majority in the high .80s. Also, the GSE scale is reported to be unidimensional. The criterion-related validity indicates positive coefficients are associated with self-efficacy related optimistic emotions and confident dispositions, whereas negative coefficients are assosicated with anxiety, depression, and stress [22].

The demographic questionaire and all three assessments, as per the previously mentioned instruments, were made at: 1) day 1 of the study (four days prior to surgery = T1) by the RA as a baseline; 2) day 5 of the study (directly following the use of intervention and prior to surgery = T2) by the RN caring for the patient; 3) day 5 of the study (directly following use of intervention and surgery = T3) by the RN caring for the patient; 4) day 10 of the study (follow-up visit = T4) by the RA when meeting with the patient. The control group participants filled out the demographic questionaire and the SAI, NRS, and GSE prior to surgery and the same instruments following surgery. Measurements and times in the study are illustrated in Table 2.

**Table 2:** **Measurements and times in study**

	**T1**	**T2**	**T3**	**T4**
**Demographics**	X*	X**		
**State Anxiety Inventory (SAI): anxiety**	X	X	X	X
**Numeric Rating Scale (NRS): pain**	X	X	X	X
**General Self-Efficacy Scale (GSE): self-efficacy**	X	X	X	X

*Note,* X = all groups (intervention & control); X* intervention groups; X** control group; T1 = day 1 baseline; T2 = immediately prior to surgery; T3 = immediately following surgery; T4 = day 10 follow-up.

### Randomization

The RAs recruited pre-scheduled SDS patients by telephone and explained the aim of the study. Once the patient provided oral consent to participate in the study per telephone, the RA arranged to meet the intervention participants four days prior to the scheduled surgery date. Participants in the control group were instructed to arrive at the SDS ward on the date of surgery to sign consent forms and fill out the outcome measurements. When the RA met with the intervention participants the RA randomly assigned the participants to a treatment group. The RA and intervention participant were blinded to the treatment until meeting on day 1 and simultaneously opening the mobile technology containing the treatment. No one was blinded after the assignment and opening of the intervention. The only similarity of the interventions were the mobile technologies used (iPods and iPads). The trial ended due to the end of the PI’s scholarship in Iceland.

### Registration

This pilot RCT was registered at the *Personuvernd* (Data Protection Authority of Iceland; #S5862) before the commencement of any data collection. http://www.personuvernd.is/information-in-english/. In addition the study was registered at ClinicalTrials.gov with an Identifier: NCT02236455/September 4, 2014.

### Ethics statement

A risk-benefit analysis was made at the time of the design of the study and was included in the application to the Institutional Review Board of the Protection of Human Subjects (IRBPHS) at the University of San Francisco (USF) and the Bioethics committee of the National University Hospital of Iceland (Landspítali). The participants were informed: 1) little to no risk was involved in partaking in the study; 2) extremely high-audio volume levels while using headphones is not advised; 3) if assigned to the ART group, avoid using the audio recording while driving an automobile, riding a bicycle, and/or operating machinery because it may make you feel drowsy; 4) individual decisions to participate or not participate in the study will not in any way affect current or future medical treatment; 5) particpants could leave the study at any given time; 6) all collected data will be assigned a unique numerical code; and 7) all written documents will be stored in a locked cabinet in the PI office for six months and then shredded. Upon discharge from the hospital, all participants received the ART recording, via a compact disc, for participating in the study. The study was approved by the IRBPHS at the USF on October 3, 2011 (#11-094) and the Bioethics Committee of the National University Hospital of Iceland (Landspítali) Reykjavik, Iceland on March 14, 2012 (#39/2011). The clinical trial number was obtained on September 9, 2014 (#NCT02236455).

### Statistical analyses

Statistical analyses were performed using the Statistical Package for the Social Sciences (SPSS) for Windows, version 21. Descriptive statistics were conducted and the research questions were analyzed by matched-pairs T-tests and ANOVA. The target sample size was 85 participants based on the power analysis done at 80% power for a five-group ANOVA with a medium effect size. Set a priori, the level of significance for statistical tests was alpha = .05 (two tailed).

## Results

### Research question 1: state anxiety

**Figure 3:**
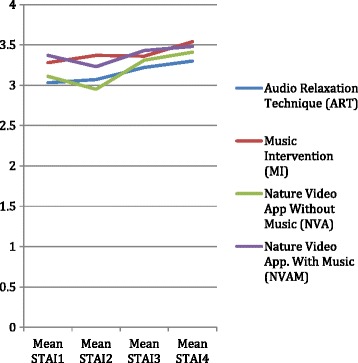
**Anxiety results for intervention groups.**

The mean anxiety scores and standard deviations (SD) for the experimental and control groups are provided in Table 3, with graphic illustration in Figure 3. According to the matched pair *t*-Test results, all experimental groups, did not have significant changes in anxiety scores from day 1 to pre-op. However, NVA and NVAM participants reported a non-significant decrease in anxiety from day 1 to pre-op. From pre-op to PO, the NVA group showed significantly higher anxiety scores (t = 2.714, df = 13, *p = .018*). Furthermore, the matched pair *t*-Test revealed all intervention groups from pre-op to day 10 had a significant increase in measured anxiety levels with the MI group (t = 1.715, df = 23, *p* = .100) being an exception. An ANOVA found no statistically significant differences in anxiety scores measured by the SAI between the five groups at any measurement point (*F =* 0.76; *p =* 0.55).

**Table 3:** **Anxiety data: group descriptives**

	**Group assignment**	**Mean**	**SD**	**N**
SAI T1 Anxiety	ART	3.03	0.51	25
	MI	3.28	0.42	25
	NVA	3.11	0.64	15
	NVAM	3.37	0.35	16
	Control	NA	NA	NA
	All Participants	2.23	2.66	82
SAI T2 Anxiety	ART	3.07	0.53	24
	MI	3.37	0.35	25
	NVA	2.95	0.73	14
	NVAM	3.23	0.53	16
	Control	2.86	0.70	24
	All Participants	3.10	0.95	103
SAI T3 Anxiety	ART	3.22	0.60	24
	MI	3.36	0.37	24
	NVA	3.31	0.52	14
	NVAM	3.43	0.39	16
	Control	3.21	0.69	18
	All Participants	3.30	0.53	96
SAI T4 Anxiety	ART	3.30	0.54	25
	MI	3.54	0.32	24
	NVA	3.41	0.44	15
	NVAM	3.48	0.27	15
	Control	NA	NA	NA
	All Participants	3.43	0.42	80

*Note.* SAI = State Anxiety Inventory, 20-items; NA = not assessed; other abbreviations as in Table 2.

### Research question 1: perceived pain levels

**Figure 4:**
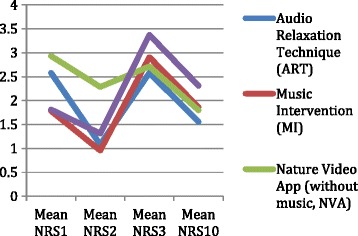
**Pain results for experimental groups.**

The mean pain level scores and SD for the experimental and control groups are provided in Table 4, with graphic illustration in Figure 4. The matched pair *t*-Test results illustrate all intervention groups’ show a dip in pain levels from day 1 to pre-op, with the ART group (t = 2.71, df = 24, *p* = .01) and the NVAM group (t = 2.45, df = 15, *p* = .03) having significant decreases in pain. All groups reported a significant increase in pain levels from pre-op to PO with the NVA group having non-significant changes in pain levels (t =1.000, df =13, *p* = .336). From pre-op to day 10, the matched pair *t*-Test results illustrate all intervention groups not having significant changes in pain levels except the MI group demonstrating a significant change (t = 2.094, df = 21, *p* = .049). By looking at Figure 4 it is clear all the intervention groups had a decrease in pain from PO to day 10 with the ART group (mean = 3.30; sd = 0.54) reporting the lowest mean level of pain. The ANOVA results indicate no statistically significant differences in pain scores measured with the NRS between the five groups at any measurement point (*F = 0.76*; *p =* 0.25).

**Table 4:** **Pain data: group descriptives**

	**Group Assignment**	**Mean**	**SD**	**N**
NRS T1 Pain	ART	2.58	2.92	25
	MI	1.78	2.24	23
	NVA	2.93	3.01	15
	NVAM	1.81	2.51	16
	Control	NA	NA	NA
	All Participants	2.23	2.66	80
NRS T2 Pain	ART	1.08	1.99	25
	MI	0.96	1.46	24
	NVA	2.29	2.84	14
	NVAM	1.31	2.24	16
	Control	2.25	2.63	24
	All Participants	1.52	2.25	103
NRS T3 Pain	ART	2.58	2.70	25
	MI	2.90	2.07	22
	NVA	2.71	2.95	14
	NVAM	3.38	2.06	16
	Control	2.74	2.38	19
	All Participants	2.84	2.41	96
NRS T4 Pain	ART	1.56	1.66	25
	MI	1.86	1.67	22
	NVA	1.80	2.71	15
	NVAM	2.31	1.82	16
	Control	N/A	N/A	N/A
	All Participants	1.82	1.92	79

*Note.* NRS = Numeric rating scale, 0–10; NA = not assessed; other abbreviations as in Table 2.

### Research question 1: perceived self-efficacy

**Figure 5:**
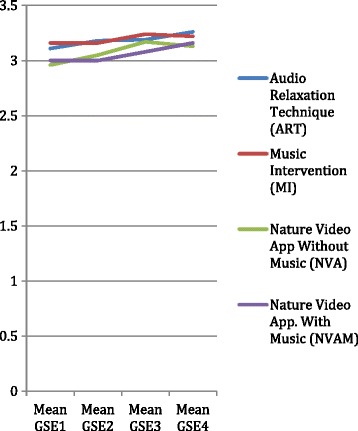
**Self-efficacy results for intervention groups.**

Experimental and control group participants’ perceived self-efficacy (SE) mean scores and SD are provided in Table 5 and, a graphic interpretation in Figure 5. According to the matched pair *t*-Test results, all intervention groups had non-significant changes in mean SE scores from day 1 to pre-op. However, the participants in the MI group (t = 2.056, df = 22, *p* = .052) and the NVA group (t = 2.409, df = 12, *p* = .033) had significant increases in SE from pre- to PO assessment. In addition, the ART (t = 2.077, df = 22, *p* = .05) and NVAM (t = 2.644, df = 14, *p* = .019) participants reported significant increase in SE from pre-op to day 10 follow-up. The control group participants’ (t = 3.674, df = 16, *p* = .002) demonstrated a significant increase in self-efficacy from pre- to PO, as well. By viewing Figure 5 the NVAM group had a steady climb in SE scores from the pre-op period and the ART had a bump in SE scores from PO to day 10. ANOVA results gleen no statistically significant differences in SE scores measured with the GSE between the five groups at any measurement point (*F = 0.16*; *p =* 0.95).

**Table 5:** **Self-efficacy data: group descriptives**

	**Group Assignment**	**Mean**	**SD**	**N**
GSE T1 Self-efficacy	ART	3.11	0.39	25
	MI	3.16	0.40	25
	NVA	2.96	0.41	15
	NVAM	3.00	0.33	16
	Control	NA	NA	NA
	All Participants	3.08	0.39	82
GSE T2 Self-efficacy	ART	3.18	0.35	23
	MI	3.16	0.37	25
	NVA	3.05	0.40	15
	NVAM	3.00	0.40	16
	Control	3.06	0.40	24
	All Participants	3.10	0.38	103
GSE T3 Self-efficacy	ART	3.19	0.38	23
	MI	3.24	0.35	23
	NVA	3.17	0.41	13
	NVAM	3.08	0.37	16
	Control	3.15	0.42	18
	All Participants	3.17	0.38	93
GSE T4 Self-efficacy	ART	3.26	0.38	25
	MI	3.22	0.39	24
	NVA	3.13	0.42	15
	NVAM	3.16	0.39	15
	Control	NA	NA	NA
	All Participants	3.20	0.39	80

*Note.* GSE = General self-efficacy scale, 10 items; NA = not assessed; other abbreviations as in Table 2.

### Research question 2

ANOVA tests were used to determine if there were statistically significant differences among the five groups (N = 105) at the various times with respect to anxiety, pain, and self-efficacy. For state anxiety, statistically significant differences were found among the groups pre-op (*F = 2.60; df = 4,98; p = .04)*, but not at day 1, PO, or day 10 follow-up. Post-hoc analyses were performed to further determine statistically significant differences at pre-op for anxiety. The post-hoc analysis for anxiety showed the MI and control groups having the greatest difference in measured anxiety, with a *p* = .06, approaching, but greater than the statistically significant level. The post-hoc analysis for pain and self-efficacy failed to indicate any specific pair of intervention groups having a statistically significant difference between them.

### Research question 3

#### Effects on anxiety characteristic

Participants who reported medical histories for anxiety and pain, with and without medication, were compared against those who reported no medical history for anxiety and pain, with and without medication. All five groups (N = 105) were combined for this analysis, as comparing participants within groups would require a much larger sample size to be statistically reliable. Matched pair *t*-Test results showed significant decreases in anxiety for those participants reporting a medical history of anxiety from pre-op to day 10 (t = −3.35, df = 20, *p* = .003). Aforementioned test results showed significant decreases in anxiety for those participants reporting no medical history of anxiety from pre-op to PO (t = −2.20, df = 67, *p* = .03) and pre-op to day 10 (t = −3.74, df = 56, *p* = .001). Matched pair *t*-Test results showed significant decreases in anxiety for those participants reporting prior use of medication for anxiety from pre-op to day 10 (t = −3.13, df =16, *p* = .007). And, for those reporting no use of medication for anxiety, there were statistically significant results for both pre- to PO (t = −2.70, df = 75, *p* = .01) and pre- op to day 10 (t = −3.99, df = 62, *p* = .001).

#### Effects on pain characteristic

Matched pair *t*-Test results showed significant decreases in pain for those participants reporting a medical history of pain from pre-op to PO (t = 2.65, df = 27, *p* = .01). This could be seen as a reason to use CM as an adjunct for individuals who report a history of pain. The same statistical test results showed significant decreases in pain for those participants reporting no medical history of pain from pre-op to PO (t = −5.82, df = 65, *p* = .001) and pre-op to day 10 (t = −3.27, df = 56, *p* = .002). Those reporting use of pain medication prior to the study had a statistically significant decrease in pain levels from pre- to PO (t = −2.65, df = 27, *p* = .01). Moreover, the matched pair *t*-Test results showed significant decreases in pain for those participants reporting no prior use of pain medication at the pre- to PO (t = −5.82, df = 65, *p* = .001) and pre-op to day 10 (t = 3.27, df = 56, *p* = .002) times.

## Discussion

This trial was piloted with the aim of looking at the feasibility and effect of four specific CT methods delivered via MT on SDS patients’ reports of state anxiety, pain levels, and perceived self-efficacy in healing. The results of the study indicate no statistical differences in the patients’ state anxiety, self-reported pain levels, or perceived SE between the groups.

### Anxiety

In regards to anxiety scores, as reported though the use of the SAI, there were no significant changes in anxiety scores for any of the groups from day 1 to pre-op. However, the participants in the NVAM (n = 15) and NVA (n = 16) groups had a non-significant decrease in anxiety from day 1 to pre-op, whereas the other intervention groups had a steady increase in anxiety over the entire time period. Therefore, the novel use of nature videos with music and without music may have had a statistically detectable positive effect on anxiety if the sample size were larger. From pre-op to PO the participants in the MI group (n = 25) reported almost the same scores and the same participants did not have significant changes in anxiety from pre-op to day 10. Allred and colleauges [3] found statistical findings within groups to support the use of music as an intervention to decrease anxiety. The other participant groups had a significant increase in anxiety regardless of the intervention. Specifically, the NVA group participants experienced significant increases in anxiety from pre-op to PO. In the NVA group (n =16) four participants reported a history of anxiety with the same number reporting use of anti-anxiety medication and, therefore the prior history may have played a role. Of the 16 participants, seven reported prior use of CT and therefore this could be an influencing factor or bias in the initial use of the nature videos. The increase in the state anxiety scores from pre-op to PO makes sense; however, one would think anxiety levels would level out from pre-op to day 10. Stress-related factors following surgery or the pre-disposition to anxiety could have influenced the statistically significant increase in reported anxiety levels from pre-op to day 10. In regards to the statistically significant differences among the five groups at the various times with respect to anxiety, pain, and self-efficacy, there was significance immediately pre-op for only the anxiety outcomes. A post-hoc analysis indicated the only pair of groups whose difference approached the .05 alpha level was the MI and control groups, with a difference whose *p* -value was 0.06.

### Pain

Self-reported pain levels, using the NRS, decreased from day 1 to pre-op, with the ART (n = 25) and NVAM (n = 15) groups reporting a significant reduction in pain. Once again, it is worth mentioning the nature video application with music delivered via an iPad had a significant effect. This is in keeping with the NVAM group showing a decline in anxiety at the same time during the study, day 1 to pre-op. Furthermore, of the 25 ART participants, one reported a history of acute pain and three reported a history of chronic pain and, four reported use of pain medication. Thirteen of the ART participants reported prior use of ART before the study and this could be an influencing factor on the positive findings for the ART group. The NVAM participants reported similar histories of pain and pain medication use and this is a minimal number reporting a history of pain and therefore the interventions may have had a positive effect on the reduction of pain from day 1 to PO due to the minimal history of pain or use of pain medications reported by the participants. Of the NVAM participants, three reported prior use of CT and since this was a novel use of nature videos in reducing pain, it is something to pay attention to for future studies since nature videos have not been known in the literature to reduce pain.

As would be expected, all groups had a significant increase in pain from pre-op to PO, however, the NVA group did not have a significant increase. The highest mean pain level PO was 3.38 (sd = 2.06) reported by the NVAM group. On a scale of 0 – 10, a report of “3.38” and even a score of “5.44” indicates a “moderate” intensity of pain using the NRS. Pain is subjective and since pain medication or the use of anti-inflammatory medication was not tracked during the study, as originally planned, it is challenging to say if the interventions had an effect from pre-op to PO, but one may say they did not increase the pain levels from day 1 to pre-op nor from PO to day 10. The use, nor specific type of pain medication, was tracked for all participants, however, the RAs anecdotally reported participants did not receive heavy doses of opiods throughout the study. The participants in the MI group (n = 25) reported significant changes in pain from pre-op to day 10 follow-up. Two of the MI group participants indicated a prior history of acute pain; four reported chronic pain; five stated use of pain medications; and five reported prior use of MI. The aforementioned history for the MI group does not appear to have a correlation with the MI group having a significant change in pain from pre-op to day 10. Sendelbach and colleagues [27] looked at the effects of music and rest on cardiac surgery patients’ post-operative pain and anxiety and found statistically significant reduction in anxiety and pain in the music group. Music is a CM that has been reported to have significant positive effects on patients’ pain and anxiety levels [2-4]. Perhaps with a larger sample size the MI group, pre- to post-op, would demonstrate a reduction. The MI participants reported the lowest mean pain score of all groups at day 1 and at the time of admission to the SDS ward. This could be a result of the type of medical condition they experienced or perhaps the MI did have a calming effect on the perception of pain from day 1 to pre-op.

### Self-efficacy

In regards to self-efficacy, as measured by the GSE, the results of the study indicate no statistical differences in the patients’ perceived self-efficacy between the groups from day 1 to pre-op. However, participants in the MI (n = 25) and NVA (n = 16) groups had a significant increase in SE from pre- to post-op and participants in the ART (n = 25) and NVAM (n = 15) groups had a significant increase in SE from pre-op to day 10 follow-up. This is a novel study in regards to the use of nature scenes delivered via an iPad for SDS patients coping with anxiety, pain, and self-efficacy in healing. This significant finding of an increase in SE for the ART group and NVAM over a period of six days is to be noted and further investigated. Overall, 33 participants (31%) of the 105 participants enrolled in the study reported a prior use of CM. This indicates the Icelandic adult population is open to complementary and alternative ways of healing. This number of participants may have swayed the approached population to participate in this study.

### Anxiety and pain characteristics

Looking at combined participants’ (N = 105) characteristic variables of self-reported anxiety and pain from the demographic questionnaire, one sees a significant decrease in anxiety from pre-op to day 10 for those reporting a history of anxiety. There was also a decrease in anxiety levels for those participants reporting no medical history of anxiety from pre-op to PO and pre-op to day 10. Participants reporting prior use of anti-anxiety medications and those reporting no prior use of medication demonstrated significant decreases in anxiety from pre-op to day 10 and, those stating no prior use of anti-anxiety medication had significant reduction in anxiety from pre-op to PO. When all the participants were combined, a significant reduction in anxiety, during the post-operative healing period, regardless of the history of taking anti-anxiety medication was identified. Of course other factors, such as sleep, nutrition, and exercise were not controlled for in this study. Looking at these combined results, it is possible to hypothesize all four interventions used in this study could play a role in reducing anxiety in SDS patients.

Self-reported pain is subjective and in pre- and post-surgical situations there are many factors playing a role in a patient’s pain level, such as anxiety and self-efficacy in healing and functioning following surgery. A limitation of this study is not correlating pain medication use prior to the surgical date, on the day of surgery, or during the five days post-operatively. Given this limitation, critics may argue the effectiveness of the interventions on a participant’s pain level. Also, prior use of pain and anti-anxiety medication by the control group participants may be over-represented compared to the other groups by a small margin. Due to the small sample size this over-representation may have impacted the participants’ responses.

Since withholding pain medication or providing a placebo is not ethical, it is challenging to conduct a study that eliminates the effects of anti-inflammatories or opiods to illuminate the effects of the CM. However, if one purely looks at the statistical significance of the outcomes per the reported history of pain or no pain, as well as the use of medication for pain or not, it is interesting to see reported decreases in pain for those participants (n = 28) reporting a medical history of pain from pre-op to PO time. And, for those participants reporting no prior history of pain, having a significant decrease in pain pre- to PO and pre-op to day 10 follow-up. Furthermore, the participants who did not use pain medication prior to the study had significant decreases in pain levels pre- to PO and pre-op to day 10 follow-up. One could speculate the participants not using pain medication prior to the study may not be dependent upon the use of pain medication for various reasons and not request it at the time of surgery, thereby allowing the CT to have more of an effect. However, one could follow-up with a correlational study and closely follow the use of pain medication pre- and PO with the use of the CT.

### Limitations

There are several limitations of this study. One limitation is the aforementioned lack of tracking medication use by the participants pre-, intra- and post-operatively. This is a challenging factor when determining the actual effects of CT on anxiety and pain reduction, because it is unclear how much influence the pain medication has on the self-reports of pain. Sample size was based on a small to medium effect size (0.20) and therefore an increase in sample size may have provided different outcomes. Another limitation was the inconsistency of the nursing staff pre- and post-operatively since the RAs were not always available to work with all of the participants upon admission to the SDS ward. Some nursing staff could be prone to administering pain medication more liberally in anticipation of pain instead of waiting for the pain to reach a certain level and then medicating for the patient’s pain. Also, since the control group participants did not answer the baseline questions at T1 when the intervention group participants did, there is a possibility completing the three assessments in the hospital on the day of surgery could have affected the overall responses. Furthermore, the free access to the popular MT may have lured the participants to take part in the study creating a bias. However, the MT are becoming more accessible to the general public today and are loosing initial novelty.

### Implications

More research using ART, MI, NVAM, and NVA as an adjunct to traditional surgical care is necessary. Using a larger, more heterogenous sample, and using the interventions over a longer period of time and at varying times during the day may provide more evidence the CT used in this study have significance between groups. Correlating the amount of time the participants use the interventions is a suggestion, in addition to closely monitoring and recording the use of pain medications. Also, using a triangulated research design may gleen other important findings to further the research of the use of CT for surgical patients. This implication is based on the anecdotal notes by the participants and provided by the RAs during this study: A female participant stated: “I am always busy and never sit down. I’m happy to have a moment to sit down and interact with the intervention.” A woman being prepared for anesthesia stated: “When I close my eyes and try to relax I see the photographs of the Icelandic landscape from the nature video application on the iPad and it really helps me to relax.” In regards to the music intervention, some participants reported wanting more of a choice regarding the type of music. Therefore, future research could allow for the participants to have a wider choice of the types of music in order to increase the appeal of listening to music as a soothing element pre- and post-operatively.

## Conclusions

In conclusion, the results of this feasibility pilot trial provided information regarding the use of CT via MT when initiated four days prior to surgery, immediately pre- and post-operatively, and five days following surgery for a homogenous Icelandic adult sample. Despite no statistically significant differences in anxiety, pain levels, or preceived self-efficacy scores between the five groups, at any measurement point, were found in this study, there were some interesting trends toward significance and generalizability for the homogenous population of Iceland. Among the group participants there were statistically significant findings, especially in the increase in perceived self-efficacy scores. And, the results indicate it is possible to use these modalities in a busy hospital setting without disruption of staff or physical complications. Based on Ecotherapy [17] and recent research on the effects of nature on the human brain [16], the NVAM show significance in the reduction of perceived pain levels from day 1 to day 5 and the NVA had a significant influence on enhancing self-efficacy from the pre- to post-operative period. The ART provided in Icelandic, showed positive effects on participants’ self-efficacy from pre-op to day 10 follow-up. The use of CT as an adjunct to reduce anxiety in individuals with a history of anxiety and taking anti-anxiety medication was a significant finding of this study when all participants were combined.

Traditionally, pharmacological agents have been used to manage anxiety and pain for pre- and post-surgical interventions. Pre- and post-surgical education, primarily conducted by nurses, has been used to increase a surgical patients’ self-efficacy in overall healing. The results of this study indicate CT when used in conjunction with traditional methods had positive influences among the groups, especially for self-efficacy. The delivery of CT via MT is a convenient method for both patients and healthcare providers because the devices may be used anytime and anywhere. The use of nature as a CT for healing the mind, body, and soul is important because appreciation of the beauty of natural surroundings deeply affects humans in a positive manner, such as evoking feelings of calm, relaxation, and contentment [28]. Therefore, continued empirical research is merited for the effects of nature in assisting peri-operative patients cope with anxiety, pain, and self-efficacy in healing. In closing, the author is very grateful for the support of the Fulbright Scholars Program, the Faculty of Nursing at the University of Iceland, and the National University Hospital of Iceland (Landspítali) in Reykjavik, Iceland.
